# Supplementary material for: Research-informed decision-making for empowering integrated care system development: Co-creating innovative solutions to facilitate enhanced service provision
Source: PLoS One. 2025 Apr 24;20(4):e0321994. doi: 10.1371/journal.pone.0321994 (PMC12021209; doi:10.1371/journal.pone.0321994)
Supplement: Attachment — Submitted filename: Response to Reviewers.docx [file pone.0321994.s003.docx]

**Response to Reviewer`s Comments**

**PONE-D-24-19911**

**Research-informed decision-making for empowering integrated care system development: Co-creating innovative solutions to facilitate enhanced service provision.**

Thank you very much for your constructive and helpful comments and suggestions for our revised manuscript. We hope you will find our changes align better with your expectations for the paper.

In response to each of the reviewer's points, we have provided detailed explanations and implemented the necessary changes directly within the manuscript using the 'Track Changes' mode in MS Word. This allows for easy identification of the modifications made unless an entire section has changed which we highlight (i.e., introduction, discussion).

**REVIEWER 1**

| **No** | **Comment** |  | **Response, Explanation and/or Revision** |
| --- | --- | --- | --- |
| **Introduction** | | | |
| **1** | The introduction is very long and rich of topics, insights, literature references and comments. I think that this could derive from the objective of the study and could cause difficulties during the papers’ reading. For this reason, I could that could be better to provide an overview of the issue, considering the background and providing relevant examples, able also to describe and highlight the literature gaps or the possible practical contributions of the study, and then defining a paragraph of literature review for the collection of the already published evidence related to the Integrated Care Management model, enlarging the already defined paragraph about ICM in the UK, not presenting only the Policy and the Practice but presenting also the literature evidence about the topic. | SP | The introduction has been revised to be more concise as per your suggestion, e.g. the history of the NHS has been moved to the appendix for readers who are interested in further details.  Although ICMs are currently gaining awareness and popularity globally, their implementation is still in its infancy. The UK is one of the few countries that has explicitly implemented integrated care systems nationally through statutory NHS organisations. Given that ICM is a central theme of the article, the authors thought it appropriate to allocate a dedicated sub-section to ICM, titled 'ICM in the UK: Policy and Practice' within the Introduction. |
| **2** | In addition, in the Introduction, the systems approach is presented and described but this part of the manuscript is not well linked to the first part of the introduction, providing some issues for the reader, in terms of knowledge and contents’ flow. | S/E | Thank you for this observation. Given that the NHS context has been moved to the Appendix in the first part of the introduction (approximately 400 words lifted from the previous version), the systems approach is now described earlier in the introduction. This ensures that the problem context is explained well enough to facilitate a smoother transition to the solution (i.e., systems approach). |
| **3** | Also, the concept of decision support tool (DST) could be better introduced and presented, if relevant from the beginning. | ED | Thank you for the recommendation. The concept of DST is introduced earlier in the introduction after having explained the systems approach as follows:  “The systems approach can be complex, and the available software’s is difficult for non-experts to use. Developing a decision support tool (DST) based on a systems approach not only makes the methodology more appealing but also enhances its practical usability. This enables end-users to interact with the system more effectively, thereby expediting the decision-making process. A DST in healthcare is a software program that aids key opinion leaders (KOLs) in making well-informed decisions by delivering pertinent and timely information. It consolidates data from multiple sources, including patient records and medical research, to provide recommendations, predict outcomes, and propose best practices. These tools help in planning by identifying trends, optimising the use of resources, and enhancing patient care, which leads to more efficient and effective healthcare services.  This article, therefore, contributes to the development of research policies for organisations aiming to advance ICM by utilising a DST that facilitates better decision-making." |
| **4** | Moreover, the Table 1, presenting some questions on how OR could achieve enhanced ICM in a hospital setting, is not so clear. Are these the research questions of the papers? Or these are some guideline questions? | ED | Thank you for raising these questions. The paragraph has been revised as follows:  “Table 1 presents a set of questions that serve as a guideline. These questions help the framework address a wide range of inquiries related to the implementation of ICM. Their global relevance lies in their ability to focus attention on the resources utilised—both financial and human—in the pursuit of patient-centred care and improved patient outcomes.” |
| **Literature Review** | | | |
| **5** | In this paragraph, I think that a summary Table could help the reader to focus on the different proposed alternatives. | UY | Thank you for the suggestion. A table that compares the alternative simulation methods is provided. |
| **Materials and Methods** | | | |
| **6** | More references may be introduced to present and justify the methodological approach and research rigour. | UY | Thank you for this recommendation. More references were provided for method justification. |
| **7** | In the section of System Modelling, more details may be provided with reference to the conducted meetings and the focus group. | UY | Thanks for pointing out this. More information about the meetings and interviews are now added to System Modelling in the Case Study section as follows:  “Semi-structured interviews and focus group meetings were carried out with specialist nurses from retinal services and managers from an ICS. The interviewees' insights and inputs allowed us to map the patient flow in the service in detail as well as the structure of the ICS. Also, inputs such as resource, capacity, and patient routing were collected via the interviews. The patient flow was verified by the nurses from the services.” |
| **8** | The section devoted to Data Collection and Analysis is general and it is difficult to understand which dataset and information are used for the study as well as how the authors collected the data. | UY | Thanks for this insight. The **Data Collection and Analysis** section guides readers about data collection and analysis steps in system modelling.  The details about the data sources and collection methods used in the study are described in the **Case Study section** where we showcased the application of the simulation model. Please see our response to the next point below. |
| **Case Study: Application of the Model to an NHS Hospital** | | | |
| **9** | The data included in the Table 2 might be moved into the Materials or could be better to clarify that this is the materials and methods section dedicated to the case, and then presenting the results. It is not clear for my point of view, how the authors collected the data and which is the data source(s). | UY | Thank you for this observation and suggestion which allowed us to elaborate on the information about data collection and sources in the case study as follows:  “To run the simulation model for the case study, we collected various data which was mainly patient level. The main data sources was the hospital, expert views and published literature/reports. The data obtained from the hospital was from a period of 36 months. The process involved comprehensive data collection and analysis due to the large size and complexity of the dataset. Working closely with the hospital’s health informatics team, we extracted relevant data from various services, including inpatient, outpatient, and emergency services. This data was used to estimate various input parameters, establish statistical distributions, and forecast future demand. Also, several statistical distributions were set to capture the variation in real life for factors such as length of stay and waiting time.  The full list of the input parameters that are used in the model is provided in Table 3 along with the source. The inputs are related to demand, human and non-human resources (such as ophthalmology consultants, nurses, and diagnostic equipment), routing rates, and treatment rates, as well as costs and revenues.” |
| **Results** | | | |
| **10** | I think that it could be better to move the scenarios’ definition and description into the methodological section. | UY | Thanks for your suggestions. The definition of the scenarios is moved to the **Case Study** section (i.e., before the Result section). |
| **Discussion** | | | |
| **11** | This section may be devoted to discuss the achieved results, and the methodological approach applied, in comparison with already published papers and reference, in order to explain the position. | ED | Thank you for the suggestion. The development of frameworks that combine both qualitative and quantitative approaches to address the challenges of healthcare within integrated care models (ICMs) using systems approaches and decision support tools is lacking. This is evidenced by the literature and our team's two decades of expertise in this field. While models have been developed to improve service planning, they have not been specifically designed for the context of ICMs, thus making like-for-like comparisons difficult. However, we have included the following in the discussion:  “Systems approaches are gaining awareness and popularity in addressing complex, messy systems through the use of simulation-based models (e.g., System Dynamics, Discrete Event Simulation, and Agent-Based Simulation) to establish the most efficient and effective delivery of services. However, there are limited examples specifically developed for the context of Integrated Care Models (ICMs). Numerous reports, studies, and research stress the need to integrate fragmented services to improve the health of the local population. Harten [52] explicitly discusses the benefits of ICMs and highlights that a firm body of evidence on the added value of transforming pathways into ICMs is hard to find, leaving room for much variation. This article addresses that major gap in the literature by providing evidence for KOLs before such changes are implemented in practice.” |
| **12** | In addition, the practical implications and the tool’s advantages for the KOL are well presented and structured, while the theoretical implications are lacking and might be presented. | ED | We appreciate your recognition of the practical implications and the structured presentation of the tool's advantages for KOLs. We understand the importance of also highlighting the theoretical implications of our work. In response to your comment, we have elaborated on the theoretical foundations and contributions of our study as follows in the discussion section.  “Our study integrates systems theory within the healthcare context, providing a comprehensive model for understanding and managing complex adaptive systems. By combining hospital and out-of-hospital services into a single framework, we advance the theoretical understanding of systems integration in healthcare. This approach underscores the importance of considering the interdependencies and interactions between different components of the healthcare system, which is crucial for developing effective ICMs. Additionally, the development and application of DSTs in our study contribute to the theoretical discourse on decision-making in healthcare.” |

**REVIEWER 2**

| **No** | **Comment** |  | **Response, Explanation and/or Revision** |
| --- | --- | --- | --- |
| **Introduction** | | | |
| **1** | The introduction was well written and effectively sets the context by discussing global healthcare challenges and the importance of integrated care. However, it could be more concise. Some sections, like the NHS's history, could be shortened to maintain reader engagement. | SP | Thank you for raising this point. The introduction has been revised to be more concise as per your suggestion, e.g. the history of the NHS has been moved to the appendix for readers who are interested in further details. |
| **Literature Review** | | | |
| **2** | The literature review nicely links the theoretical framework to practical applications in healthcare, providing relevant examples and citations, but it could benefit from a more critical analysis and a better synthesis of the literature. | ED | Thank you for this suggestion. The literature review has been updated to include a more critical analysis and improved synthesis of the literature. |
| **Results** | | | |
| **3** | Although the results section provides a thorough analysis of the impact of interventions on activity, resource utilisation, costs, and revenues, you could discuss the implications of the results here, linking back to your study's objectives and pertinent literature. | ED | Thank you for raising the need for discussing the implications of the results. The following paragraph has been added to the results section linking back to the study objectives and the literature.  “These results enable the evaluation of changes at a broader system level rather than in isolation. This approach aligns with the objectives of integrated care, which aims to bring together local partners and services—such as primary, secondary, and community care in the case of retinal services—to improve local patient services. By introducing virtual community clinics and nurse injectors, new referrals can increase, leading to early intervention that is known to improve vision and outcomes. Additionally, examining the impact on the broader system aids in reducing disparities in outcomes, patient experiences, and access to care. This comprehensive analysis also empowers key opinion leaders to improve productivity and cost-effectiveness, thereby aiding the NHS in fostering wider social and economic growth.” |
| **Discussion** | | | |
| **4** | The discussion part highlights the practical impact of the DST on decision-making and service provision in the NHS. However, a more critical perspective is needed to address potential limitations and challenges in implementing the proposed interventions. It would also be useful to discuss the generalisability of the findings to other healthcare settings or countries with similar healthcare systems. | ED | Thanks for addressing this point. The following two paragraphs have been added into the discussion to address this comment.  “However, the success of implementing virtual clinics is contingent upon having a strong digital infrastructure and ensuring that patients have access to the necessary technology, which may not be consistently available across all NHS regions. This discrepancy could worsen existing health disparities, particularly in rural or underserved areas. Furthermore, the introduction and training of nurse injectors in retinal services demand substantial investment in both education and continuous professional development to maintain high standards of patient care. There may also be resistance from current staff and potential regulatory obstacles that could hinder the quick adoption of these new roles. Additionally, the effectiveness of virtual clinics and nurse injectors relies on smooth coordination and communication across various levels of care, which is often challenging within the NHS's complex and fragmented system.  It is important to consider that these interventions hold potential for application in diverse healthcare settings or countries with comparable healthcare systems. For instance, in countries like Canada, Australia, and parts of Europe, where integrated care is prioritised amid challenges of rising demand and resource constraints, similar benefits may be realised. However, the success of these interventions in varied contexts hinges on several critical factors. Challenges observed in the NHS, such as digital infrastructure limitations, investment needs for nurse injector training, and the necessity for effective coordination across care levels, are applicable globally. Moreover, differences in cultural norms and regulatory frameworks in healthcare delivery systems necessitate careful consideration. Adaptations tailored to local practices are essential to ensure these interventions effectively address specific healthcare system needs and conditions. Therefore, while the fundamental principles of these interventions are broadly applicable, their implementation requires customisation to optimise outcomes within each unique healthcare setting.” |
