# Supplementary material for: Research-informed decision-making for empowering integrated care system development: Co-creating innovative solutions to facilitate enhanced service provision
Source: PLoS One. 2025 Apr 24;20(4):e0321994. doi: 10.1371/journal.pone.0321994 (PMC12021209; doi:10.1371/journal.pone.0321994)
Supplement: Attachment — Submitted filename: Response_to_Reviewers_auresp_2.docx [file pone.0321994.s004.docx]

**Response to Reviewer`s Comments**

**PONE-D-24-19911R1**

**Research-informed decision-making for empowering integrated care system development: Co-creating innovative solutions to facilitate enhanced service provision.**

Thank you very much for your constructive and helpful comments and suggestions for our revised manuscript. We hope you will find our changes align better with your expectations for the paper.

In response to each of the reviewer's points, we have provided detailed explanations and implemented the necessary changes directly within the manuscript using the 'Track Changes' mode in MS Word. This allows for easy identification of the modifications made unless an entire section has changed which we highlight (i.e., introduction, discussion).

**REVIEWER 1**

| **No** | **Comment** | **Response, Explanation and/or Revision** |
| --- | --- | --- |
| **1** | The paragraph "ICM in the UK: Policy and Practice" is well conceived, but I think that this paragraph is not so connected with the other introductive sections, and this should be a problem for the paper's readiness. Why do you not think to introduce the concepts and the topics of this paragraph in the first part of the Introduction and then maintain the section of the Literature review? | Thank you for this helpful suggestion. We agree that the section *'ICM in the UK: Policy and Practice'* is better integrated into either the Introduction or the Literature Review (LR) rather than presented as a standalone section. As a result, we have removed the separate section and incorporated its key concepts into the LR, using italicized headings as signposts to enhance the paper's flow and coherence. This restructuring ensures a more seamless integration of the UK policy context, highlighting its significance as one of the few countries globally to adapt the ICM approach within the broader theoretical framework outlined in the LR. |
| **2** | Table 1 allows one to clarify better the role of the proposed questions in light of the background provided but anyway, I think that also one or two more precise research question(s) for the paper, connected with the objective defined by the authors, should be proposed to help the reader understanding all the paper. | The following question is added to the table, aligning with the objectives defined and connected to the case study discussed in the paper.   - How does the implementation of community-based virtual clinics, independently and in combination with nurse-led services, affect patient waiting times and resource utilisation in hospital-based specialty services compared to baseline operations? |
| **3** | Considering that the authors stated in the methodological section that semi-structured interviews and focus groups were conducted, I think that some details regarding the questions posed or the information/areas investigated should help. | We thank the reviewer for their valuable feedback and have enhanced our methodology section to provide more detailed information about stakeholder engagement and the iterative co-creation process.  The following has been added to “*System Modelling in the Case Study*” section of the article.  “The co-creation process involved extensive collaboration with key stakeholders from the hospitals retinal services, including consultant ophthalmologists, specialist nurses, and the service manager. This engagement was structured through three comprehensive rounds of semi-structured interviews and focus groups, each serving a distinct purpose in the model development process.  The first round focused on understanding and mapping the current patient pathways. Through semi-structured interviews, we explored:   - What is the complete patient journey from referral to discharge? - What are the key decision points throughout the pathway? - What resources (human and equipment) are required at each stage? - What are the typical treatment times for different procedures? - How do patient flows vary between different conditions and treatments?   The second round concentrated on verification and detailed data collection. Stakeholders were presented with initial pathway mappings and asked to verify and enhance them. Key questions included:   - Are these pathway representations accurate and complete? - What specific resources are consumed at each touch-point? - What variations exist in treatment times between different patient types? - What factors influence clinical decision-making at each stage? - What are the current bottlenecks and resource constraints?   The final round focused on model validation and scenario development. Stakeholders were presented with the complete pathway mapping and preliminary Simul8 model. Questions explored:   - Does this model accurately represent your service operations? - Are there any missing elements or inaccuracies in the model? - What scenarios would be most valuable to test? - What metrics would be most useful for evaluating different scenarios?   Each round of stakeholder engagement led to specific refinements in the model. The iterative nature of this process ensured that the final simulation model accurately represented the real-world operation of the retinal service. All stakeholder interactions were thoroughly documented through meeting minutes, pathway diagrams, and data collection sheets, ensuring transparency and traceability in the model development process.  This structured approach to stakeholder engagement was crucial in developing a model that not only accurately represented the current service but also enabled meaningful scenario testing for service improvement.” |
| **4** | Another important point that I think is missing also in this updated version of the paper is related to the comparison of the presented study with other already published evidence. How does the paper differ from or confirm previous evidence? | We have addressed this by adding the following paragraph to the discussion section that explicitly positions our work within the existing literature. This addition highlights how our comprehensive DES model for healthcare services within an integrated care framework differs from previous studies that typically focused on isolated aspects of services. We emphasise the novel contribution of our work in creating a holistic modelling approach that captures the entire patient pathway, multiple service points, and resource interactions within a single framework - an approach not previously implemented in healthcare services modelling. We believe this addition strengthens the paper by clearly articulating its unique contribution to the field.  While previous studies have explored DES in healthcare settings, our work presents the first comprehensive DES model specifically designed for healthcare services within an integrated care framework. For instance, unlike existing models that typically focus on isolated aspects of ophthalmology services [61], our approach uniquely captures the entire patient pathway, incorporating multiple service points, resource interactions, and care transitions within a single modelling framework. This holistic approach, embedded within a DST environment, enables system-wide analysis and optimisation that wasn't previously possible with more narrowly focused models. This comprehensive integration of services within a single simulation framework represents a novel contribution to both healthcare modelling and ophthalmology service improvement literature. |

**REVIEWER 2**

| **No** | **Comment** |  | **Response, Explanation and/or Revision** |
| --- | --- | --- | --- |
| **1** | The manuscript still lacks detailed information on the extent and nature of stakeholder involvement in the co-creation process with hospital staff. Providing more specifics about stakeholder engagement, the feedback process, and how their input influenced the development of the decision support tool (DST) would enhance the study's validity and relevance. | ED | We thank the reviewer for their valuable feedback and have enhanced our methodology section to provide more detailed information about stakeholder engagement and the iterative co-creation process.  The following has been added to “*System Modelling in the Case Study*” section of the article.  “The co-creation process involved extensive collaboration with key stakeholders from the hospitals retinal services, including consultant ophthalmologists, specialist nurses, and the service manager. This engagement was structured through three comprehensive rounds of semi-structured interviews and focus groups, each serving a distinct purpose in the model development process.  The first round focused on understanding and mapping the current patient pathways. Through semi-structured interviews, we explored:   - What is the complete patient journey from referral to discharge? - What are the key decision points throughout the pathway? - What resources (human and equipment) are required at each stage? - What are the typical treatment times for different procedures? - How do patient flows vary between different conditions and treatments?   The second round concentrated on verification and detailed data collection. Stakeholders were presented with initial pathway mappings and asked to verify and enhance them. Key questions included:   - Are these pathway representations accurate and complete? - What specific resources are consumed at each touch-point? - What variations exist in treatment times between different patient types? - What factors influence clinical decision-making at each stage? - What are the current bottlenecks and resource constraints?   The final round focused on model validation and scenario development. Stakeholders were presented with the complete pathway mapping and preliminary Simul8 model. Questions explored:   - Does this model accurately represent your service operations? - Are there any missing elements or inaccuracies in the model? - What scenarios would be most valuable to test? - What metrics would be most useful for evaluating different scenarios?   Each round of stakeholder engagement led to specific refinements in the model. The iterative nature of this process ensured that the final simulation model accurately represented the real-world operation of the retinal service. All stakeholder interactions were thoroughly documented through meeting minutes, pathway diagrams, and data collection sheets, ensuring transparency and traceability in the model development process.  This structured approach to stakeholder engagement was crucial in developing a model that not only accurately represented the current service but also enabled meaningful scenario testing for service improvement.” |
| **2** | Furthermore, the manuscript would benefit from a more thorough discussion of its limitations, including challenges related to data collection and potential biases in stakeholder engagement. Addressing these limitations would provide a more balanced perspective on the study's contributions and scope. | ED | The following paragraph is incorporated into the discussion of limitations and potential biases in stakeholder engagement.  “The complexity of stakeholder engagement across multiple organisations also presented challenges. While we engaged with key stakeholders from the retinal services, achieving comprehensive representation from all touchpoints in the patient pathway (including primary care and community services) proved challenging. This limitation potentially affects the model's ability to capture all nuances of inter-organizational interactions and their impact on patient flow. Furthermore, stakeholder availability constraints meant that some validation sessions had to be conducted with partial representation, which could introduce bias in the model's assumptions and structure. The varying levels of technical expertise among stakeholders also influenced their ability to critically evaluate certain aspects of the model, particularly its technical components.  The following is incorporated discussing the challenges around data collection.  For example, precise treatment times, resource utilisation rates, and detailed patient pathway variations are rarely captured systematically. This limitation becomes more pronounced when modelling integrated care pathways that span multiple organisations and care settings. In our study, while we obtained core operational data from the retinal services, granular data about patient transitions between services, detailed resource consumption patterns, and accurate waiting times at various pathway stages were not readily available. This challenge of data availability often necessitates extensive manual data collection, expert estimations, and assumptions, which could impact model accuracy and generalisability.” |
