# Supplementary material for: Research-informed decision-making for empowering integrated care system development: Co-creating innovative solutions to facilitate enhanced service provision
Source: PLoS One. 2025 Apr 24;20(4):e0321994. doi: 10.1371/journal.pone.0321994 (PMC12021209; doi:10.1371/journal.pone.0321994)
Supplement: Attachment — Submitted filename: Response_to_Reviewers_auresp_3.docx [file pone.0321994.s005.docx]

**Response to Reviewer`s Comments**

**PONE-D-24-19911R2**

**Research-informed decision-making for empowering integrated care system development: Co-creating innovative solutions to facilitate enhanced service provision.**

Thank you very much for your constructive and helpful comments and suggestions for our revised manuscript. We hope you will find our changes align better with your expectations for the paper.

In response to the reviewer's points, we have provided detailed explanations and implemented the necessary changes directly within the manuscript using the 'Track Changes' mode in MS Word. This allows for easy identification of the modifications made unless an entire section has changed which we highlight (i.e., introduction, discussion).

**REVIEWER 1**

| **No** | **Comment** | **Response, Explanation and/or Revision** |
| --- | --- | --- |
| **1** | While the manuscript addresses an important topic, key concerns remain unresolved. The study heavily relies on literature review without sufficiently synthesizing key theoretical contributions, and a clear theoretical framework is lacking. | Thank you for your thoughtful assessment of our manuscript. While we appreciate your recognition of the topic's importance, the strength of the study is the theoretical framework. Our work is firmly grounded in three interconnected theoretical pillars: Systems Thinking Theory (pages 5-7), Integrated Care Theory (pages 3-5), and Decision Support Theory (pages 8-10). Our primary theoretical contribution bridges systems thinking with practical integrated care model implementation by integrating multiple theoretical perspectives, translating theory to practice through our novel decision support tool approach, and extending methodological innovation through co-creation principles. The framework specifically addresses the gap identified by Shaw et al. (2017) regarding the need for holistic research approaches capable of reflecting healthcare environments' complexity while accommodating diverse epistemological perspectives. |
| **2** | The methodology section lacks critical details, including the number of interviews and focus groups conducted, participant selection criteria, and the thematic analysis process for qualitative data. The absence of clarity on the sampling method, number of participants, and consideration of data saturation affects the study’s transparency and reproducibility. | Thank you for highlighting this concern about our methodology. We would like to clarify that our systems thinking approach differs fundamentally from traditional qualitative research. Our purpose was not thematic analysis, but rather accurate system conceptualisation and pathway mapping with key stakeholders who possess expert knowledge of the retinal services system. As described on page 19, we conducted three comprehensive rounds of semi-structured interviews and focus groups with consultant ophthalmologists, specialist nurses, and the service manager - individuals with the authority and expertise to validate the entire patient pathway. Each round served a specific purpose: initial mapping, verification with detailed data collection, and model validation with scenario development. This targeted approach with subject matter experts is consistent with established practice in operations research and systems modelling, where the emphasis is on accurate representation of complex systems rather than qualitative saturation. The resulting model was validated through statistical comparison with observed data, demonstrating high accuracy within a 5% margin. |
| **3** | The ethics statement mentions that "no ethics approval was required as the study did not involve human subjects," yet interviews and focus groups were conducted. | We apologise for the oversight here. University of Hertfordshire ethical approval was obtained for this study. To clarify, no patient personal information was collected or stored at any stage of the research. For all interviews and workshops, formal participant consent was obtained from healthcare professionals, with clear information provided about data usage, storage protocols, and confidentiality measures in accordance with institutional requirements. The systems modelling approach focused exclusively on anonymised operational data and expert knowledge of patient pathways.  *Page 22 in the manuscript regarding ethics has been updated accordingly, i.e., University ethical approval was obtained for this study. For all interviews and workshops, formal participant consent was obtained from healthcare professionals, with clear information provided about data usage, storage protocols, and confidentiality measures in accordance with institutional requirements.* |
